# Genome-Wide DNA Polymorphisms in Seven Rice Cultivars of *Temperate* and *Tropical Japonica* Groups

**DOI:** 10.1371/journal.pone.0086312

**Published:** 2014-01-21

**Authors:** Yuko Arai-Kichise, Yuh Shiwa, Kaworu Ebana, Mari Shibata-Hatta, Hirofumi Yoshikawa, Masahiro Yano, Kyo Wakasa

**Affiliations:** 1 Genome Research Center, NODAI Research Institute, Tokyo University of Agriculture, Setagaya, Tokyo, Japan; 2 Genetic Resources Center, National Institute of Agrobiological Sciences, Tsukuba, Ibaraki, Japan; 3 Department of Bioscience, Tokyo University of Agriculture, Tokyo, Japan; 4 Agrogenomics Research Center, National Institute of Agrobiological Sciences, Tsukuba, Ibaraki, Japan; Institute of Genetics and Developmental Biology, Chinese Academy of Sciences, China

## Abstract

Elucidation of the rice genome is expected to broaden our understanding of genes related to the agronomic characteristics and the genetic relationship among cultivars. In this study, we conducted whole-genome sequencings of 6 cultivars, including 5 *temperate japonica* cultivars and 1 *tropical japonica* cultivar (Moroberekan), by using next-generation sequencing (NGS) with Nipponbare genome as a reference. The *temperate japonica* cultivars contained 2 sake brewing (Yamadanishiki and Gohyakumangoku), 1 landrace (Kameji), and 2 modern cultivars (Koshihikari and Norin 8). Almost >83% of the whole genome sequences of the Nipponbare genome could be covered by sequenced short-reads of each cultivar, including Omachi, which has previously been reported to be a *temperate japonica* cultivar. Numerous single nucleotide polymorphisms (SNPs), insertions, and deletions were detected among the various cultivars and the Nipponbare genomes. Comparison of SNPs detected in each cultivar suggested that Moroberekan had 5-fold more SNPs than the *temperate japonica* cultivars. Success of the 2 approaches to improve the efficacy of sequence data by using NGS revealed that sequencing depth was directly related to sequencing coverage of coding DNA sequences: in excess of 30× genome sequencing was required to cover approximately 80% of the genes in the rice genome. Further, the contigs prepared using the assembly of unmapped reads could increase the value of NGS short-reads and, consequently, cover previously unavailable sequences. These approaches facilitated the identification of new genes in coding DNA sequences and the increase of mapping efficiency in different regions. The DNA polymorphism information between the 7 cultivars and Nipponbare are available at NGRC_Rices_Build1.0 (http://www.nodai-genome.org/oryza_sativa_en.html).

## Introduction

Genome sequence data have greatly increased with significant advances in next-generation sequencing (NGS) technology. Millions of DNA polymorphisms, including single nucleotide polymorphisms (SNPs) and insertion-and-deletion polymorphisms (InDels), have been obtained using this information by using high-throughput methods and bioinformatic tools. SNPs and InDels have been used as DNA markers. In particular, numerous SNPs have facilitated whole-genome genotyping and genome-wide association studies in animals and higher plants [1]–[9]. DNA markers are a powerful tool for marker-assisted breeding, quantitative trait locus analysis, and genome-wide association analyses [10], [11]. Rice is one of the most important crops worldwide; the genome sequence of 2 rice groups, *japonica* and *indica*, have been established by the International Rice Genome Sequencing Project (IRGSP) [12], [13] and the Beijing Genomics Institute [14]. To date, the whole genomes of 4 cultivars of the *japonica* group (Koshihikari, Omachi, Eiko, and Rikuu-132) have been sequenced using the NGS technology and mapped to the Nipponbare reference sequences [5], [6], [15]. Recently, by using only about 1× genome coverage, the genomes of 1,083 *indica* and *japonica* cultivars were sequenced by NGS, revealing the relationship of rice cultivars spread across wide regions [4].

NGS is also an important technology in the field of genomics. Advances in bioinformatics have facilitated the production of complete genome sequences of bacteria, viruses, and yeasts, as well as individual human chromosomes, through *de novo* assembly of short-reads sequenced by NGS [16]. However, at present, further technological developments are needed to allow complete whole genome sequencing using this method in biological species having a large genome size. Bioinformatics has been useful in genetic analyses, such as identification of mutations in functional genes, in not only microorganisms but also plants. Several bioinformatic studies on rice and *Arabidopsis* have been reported, e.g. identification of T-DNA insertion loci in activation-tagged *Arabidopsis* lines, mapping the physical location of point mutations in *Arabidopsis* genes, and mapping various transposable elements in the rice genome [17]–[19]. Bioinformatic tools have thus become important in the identification of genes.

Complete sequencing of the rice genome by NGS technology alone remains challenging, because of a low sequence homology that hinders mapping to the reference sequences in many regions. Consequently, there are gaps in the sequence of the sample genome. These unavailable sequences might contain important functional genes, which remain unknown; obtaining these unavailable sequences may facilitate more efficient use of NGS data. Such sequences might be higher in the *tropical japonica* cultivar that is considerably different from the *temperate japonica* reference cultivar [20]. Unavailable sequences have also resulted from biased sequences. Increasing the efficacy of unavailable sequences might be useful for enhancing the value of NGS data.

In this study, we sequenced 5 *temperate japonica* cultivars: Yamadanishiki and Gohyakumangoku as well as Omachi, the sequencing information for which has been previously reported, are used for sake brewing. Kameji is one of the oldest landrace like Omachi. Koshihikari and Norin 8 are modern cultivars and similar to reference Nipponbare. One *tropical japonica* cultivar Moroberekan, which is cultivated in West Africa and considered to confer durable resistance to rice blast disease [21] as well as rice yellow mottle virus [22], was also sequenced. Among the sequences of these cultivars, only those of Koshihikari and Omachi were supported by deep sequencing data [6], [15]. By sequencing various cultivars, we showed that deep sequencing over 30× the genome improved data acquisition in gene regions, particularly in the coding DNA sequences (CDSs). In addition, the use of unmapped reads obtained by assembling contigs helped in identifying new CDSs even in the cultivar Moroberekan, which showed the highest number of SNPs. Identification of more CDSs might increase the value of NGS technology.

## Results

### Whole-genome Sequencing and *in silico* Mapping

The genomic DNA of 6 cultivars, namely, Yamadanishiki, Kameji, Gohyakumangoku, Koshihikari, Norin 8, and Moroberekan, were sequenced using the NGS technology. More than 170 million short-reads from each cultivar were generated. Short-reads of Omachi that had been produced in a previous study [15] were also used in these analyses. Short-reads from the genome of each cultivar were mapped to the Nipponbare reference genome, and 81.5–94.0% and 2.2–13.4% of the total short-reads were successfully mapped to chromosomal and organelle genomes, respectively (Table S1). The short-reads that were mapped to the genome were classified into 2 groups: unique or multiple locations. The short-reads uniquely mapped to chromosomes varied from 69.7% to 83.1% of the generated reads among cultivars and corresponded to over 9.9 Gb of the Nipponbare genome (Table S1). Average sequencing depths of the uniquely mapped reads in each cultivar were from 32× to 56× across the entire genome and covered 83.4–90.1% of the Nipponbare reference genome IRGSP1.0 (Table 1). The highest coverage (%) with a sequencing depth of ≥5 was found in Koshihikari, and the lowest one was found in Moroberekan. The coverage of uniquely mapped reads suggested an increasing tendency in modern cultivars similar to Nipponbare compared to that in old cultivars and *tropical japonica* cultivar. Thus, more than 83% of the whole genome sequences of various cultivars were covered using NGS with the Nipponbare genome as reference.

**Table 1:** Coverage and sequencing depth of mapped reads with reference to the Nipponbare chromosomal genome IRGSP1.0.

	Mapped reads		Uniquely mapped reads		
	Number ofnucleotides (bp)	Genome coveragewith sequencingdepth ≥5 (%)	Number ofnucleotides (bp)	Genome coveragewith sequencingdepth ≥5 (%)	Average of sequencingdepth (fold)
Omachi	19,860,548,296	95.9	16,630,615,027	87.3	51
Yamadanishiki	14,879,630,564	96.0	12,458,183,627	88.8	38
Kameji	15,440,489,841	95.6	13,024,192,299	87.8	40
Gohyakumangoku	20,204,766,808	96.2	17,088,347,679	89.3	51
Koshihikari	16,560,127,574	97.0	13,914,811,848	90.1	41
Norin 8	22,155,138,982	97.0	18,740,487,803	89.6	56
Moroberekan	11,909,078,634	91.5	9,924,861,817	83.4	32

### Sequencing of Gene Regions using NGS Technology

**Figure 1:**
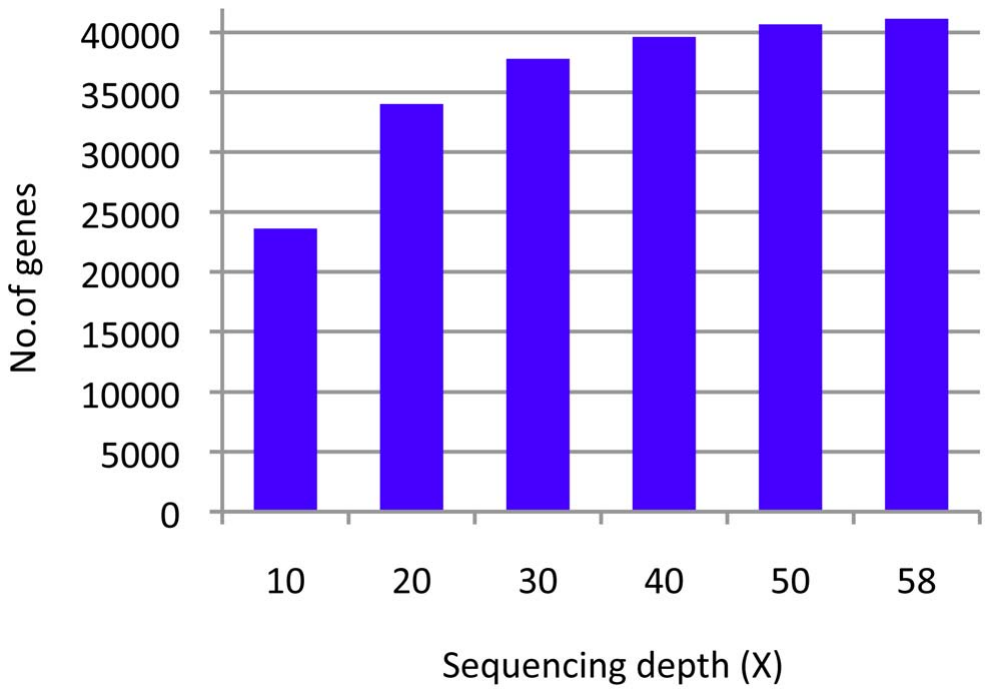
Relationships between sequencing depth and sequence coverage in the coding sequences. Omachi reads that were originally sequenced using a sequencing depth of 58× the genome were randomly eliminated to produce adjusted sequencing depths of 50×, 40×, 30×, 20×, and 10× the genome. The x- and y-axes show the sequencing depth and the number of genes covered with short-reads in over 90% of the coding sequences, respectively.

Although the coverage level described above seemed to be sufficient to obtain remarkable numbers of DNA markers, obtaining further sequences of gene regions would be ideal. CDSs of approximately 45,000 genes have been annotated in the Nipponbare reference genome IRGSP-1.0 [13]. To improve the information available for gene regions, we investigated the relation between sequencing depth and sequence coverage in the CDSs of gene regions by using the Omachi reads as a test case. Few Omachi short reads were randomly eliminated to produce various sequencing depths that corresponded to 50×, 40×, 30×, 20×, and 10× coverage of the genome. The short-reads of each set were mapped onto the Nipponbare reference genome, and the coverage of the CDSs was calculated. Figure 1 shows the number of genes that were covered by short-reads in 90% of the regions. A 90% limit was used to account for possible deletions in the CDSs. In all, 23,616 genes had 90% or more of the CDS covered by short-reads when the sequencing depth was 10× the genome; this was only approximately half of the genes in the genome (Figure 1). When the sequencing depth was 20× the genome, the number of genes obtained was 1.44 times as that obtained with a sequencing depth of 10× the genome. When the sequencing depth was increased to 30× the genome, the number of genes increased to 37,793, which was 1.6 times the number covered with a depth of 10× the genome and was equivalent to approximately 85% of the genes in the genome. The number of genes did not show any marked increase with a sequencing depth of 40× or 50×, yielding 1.05 and 1.08 times the number of genes obtained with a sequencing depth of 30× the genome, respectively. This result suggested that a sequencing depth of at least 30× the genome was required, for example, to identify a specific gene with mutations in the CDSs by using whole genome sequencing. All 7 cultivars were covered with a sufficient sequencing depth for future analyses of genes by using this criterion (Table 1).

However, the genomes of the 7 cultivars harboured different number of CDSs (Table 2). In the 7 cultivars, most genes showed 100% coverage of CDSs and those with CDSs covered in excess of 80% accounted for >90% of the total number of genes. The maximum and minimum numbers of genes with completely covered CDSs was 40,296 in the Koshihikari and 33,229 in Kameji cultivars, respectively; the sequencing depths for these cultivars were 41× and 40× the genomes, respectively. The sequencing depth of the Moroberekan genome (32×) was the lowest, and CDSs of 1,188 genes were not covered by any short-reads; this number was more than twice compared to that in other cultivars. However, in this genome, the number of genes with 100% coverage of CDSs was 39,193, which was the second highest number of genes after Koshihikari. These results indicated that, in more than 90% of the total genes, >80% of the CDSs could be determined by using whole genome sequencing with a sequencing depth of 30× the genome.

**Table 2:** Numbers of genes with defined sequence coverage in each cultivar.

Sequence coverage (%)*	Omachi	Yamadanishiki	Kameji	Gohyakumangoku	Koshihikari	Norin 8	Moroberekan
100	36,629	36,687	33,229	38,940	40,296	38,589	39,193
90≤	4,522	3,669	5,861	2,674	2,039	3,333	2,324
80≤	1,115	1,326	1,897	822	515	742	595
70≤	480	743	1,054	432	259	366	258
60≤	321	441	599	254	183	216	196
50≤	211	273	446	184	152	173	166
40≤	165	233	283	160	122	136	128
30≤	121	169	185	135	94	108	124
20≤	127	143	165	121	119	111	106
10≤	108	102	126	76	67	90	118
0<	95	107	124	92	80	83	118
0	620	621	545	624	588	567	1,188

Sequence coverage indicating the percentage of coding sequences covered by short-reads.

### Comparison of SNPs and InDels among the Different Cultivars and the Nipponbare Genome Sequences

SNPs and InDels were detected when the sequence of each cultivar was compared with the Nipponbare reference genome sequences IRGSP1.0 (Table 3). The numbers of SNPs detected in the cultivars in a descending order are as follows: Moroberekan (827,448), Gohyakumangoku (142,087), Omachi (132,799), Yamadanishiki (123,657), Koshihikari (115,455), Norin 8 (85,291), and Kameji (76,967). Moroberekan showed 10-fold more SNPs than those found in Kameji. The number of SNPs with a call rate of >90% was proportional to that of insertions and deletions in those cultivars, except for Kameji (Table 3). The number of SNPs observed in the Kameji genome was about half the number expected on the basis of the number of insertions and deletions. False-positive SNPs were eliminated by filtering out SNPs with a call rate of <90%. When the call rate of >70% was used, the number of SNPs was almost equal to that expected from the number of insertions and deletions in Kameji (Figure S1). Although the cause for this phenomenon remains unknown, approximately 80,000 SNPs obtained are usable as DNA markers (Table 3). The quality of the detected SNPs was evaluated by analysing the genome of each cultivar using SNP array technology. The SNP validation rates reached >99.5% (Table S2), indicating that the SNPs could effectively be used as DNA markers.

**Table 3:** Densities of SNPs and InDels on individual chromosomes detected between the various cultivars and the Nipponbare genome IRGSP1.0.

	Chr.	Omachi	Yamadanishiki	Kameji	Gohyakumangoku	Koshihikari	Norin 8	Moroberekan
SNPs								
	1	14,683	14,460	6,217	7,059	18,138	2,780	82,017
	2	10,198	9,322	7,203	8,876	6,603	5,893	41,374
	3	6,111	6,074	5,428	4,138	4,499	2,920	50,560
	4	26,042	16,178	14,865	25,723	17,242	22,211	84,996
	5	5,706	6,119	2,677	5,801	2,053	1,184	61,350
	6	15,461	15,329	3,307	13,571	2,019	6,778	94,539
	7	12,938	10,644	10,752	10,431	11,829	3,136	61,282
	8	10,751	15,596	4,995	6,727	11,820	2,137	85,159
	9	2,953	1,379	1,522	17,471	547	2,700	38,306
	10	4,460	6,663	3,627	4,789	6,237	7,999	92,007
	11	17,834	17,610	12,215	27,182	21,715	17,399	61,069
	12	5,662	4,283	4,159	10,319	12,753	10,154	74,789
	Total	132,799	123,657	76,967	142,087	115,455	85,291	827,448
Insertions								
	1	1,900	2,037	2,236	1,328	2,312	619	8,752
	2	1,469	1,423	1,950	1,344	1065	952	4,797
	3	893	937	1,041	875	795	522	5,492
	4	3,183	2,672	3,192	3,361	2,657	2,901	7,057
	5	921	942	753	968	391	327	5,684
	6	1,526	1,618	1,038	1,640	373	858	8,267
	7	1,472	1,345	1,979	1,247	1,518	473	5,981
	8	1,248	1,586	1,482	938	1,088	337	7,017
	9	403	309	724	1,628	180	473	3,425
	10	645	823	2,419	663	848	893	6,637
	11	1,784	1,855	2,835	2,718	2,277	1,812	5,924
	12	852	725	1,002	1,284	1,618	1,321	6,031
	Total	16,296	16,272	20,651	17,994	15,122	11,488	75,064
Deletions								
	1	1,956	2,045	2,333	1,440	2,476	679	9,700
	2	1,618	1,508	2,085	1,371	1,138	978	5,454
	3	1,035	1,025	1,057	829	884	509	6,129
	4	4,065	3,404	4,085	4,248	3,477	3,772	8,586
	5	1,060	1,083	787	1,127	427	411	6,457
	6	1,731	1,814	1,078	1,712	380	967	9,479
	7	1,698	1,494	2,211	1,315	1,668	505	6,642
	8	1,448	1,741	1,597	1,007	1,126	282	7,634
	9	479	351	795	1,806	151	487	3,835
	10	773	900	2,688	743	883	945	7,541
	11	2,112	2,119	3,183	3,146	2,608	2,181	6,304
	12	1,095	897	1,158	1,577	1,844	1,477	6,772
	Total	19,070	18,381	23,057	20,321	17,062	13,193	84,533

The SNPs obtained from the 7 cultivars were annotated using the gene set of IRGSP1.0. On the basis of the average values of the 7 cultivars, 17.6±1.4% of the total SNPs and 17.4±0.7% of the total InDels were located within a gene (Table 4). Among them, 5.5±0.7% SNPs and 2.4±0.3% InDels were located in CDSs. In all, there were 3.0±0.4% non-synonymous SNPs. The percentages of SNPs and InDels belonging to each category were similar in the 7 cultivars. These data are available at a GBrowse, NGRC_Rices_Build1.0 (corresponding to IRGSP1.0) (http://www.nodai-genome.org/oryza_sativa_en.html).

**Table 4:** Annotation of SNPs, insertions, and deletions.

	Omachi	Yamadanishiki	Kameji	Gohyakumangoku	Koshihikari	Norin 8	Moroberekan
SNP							
Intergenic	107,927	100,279	62,598	119,362	95,469	69,833	700,701
Genic	24,872	23,378	14,369	22,725	19,986	15,458	126,747
Intron	12,351	11,932	7,354	11,248	9,641	7,667	68,116
UTRs	4,665	4,255	2,387	4,367	3,737	2,929	24,131
CDS	7,856	7,191	4,628	7,110	6,608	4,862	34,500
Synonymous	3,668	3,355	2,100	3,251	3,008	2,142	15,876
Nonsynonymous	4,188	3,836	2,528	3,859	3,600	2,720	18,624
Insertion, Deletion							
Intergenic	29,041	28,302	36,091	32,085	26,584	20,481	131,783
Genic	6,325	6,351	7,617	6,230	5,600	4,200	27,814
Intron	3,971	4,019	4,907	3,869	3,345	2,551	18,236
UTRs	1,515	1,488	1,652	1,488	1,381	958	6,616
CDS	839	844	1,058	873	874	691	2,962

SNPs, insertions, and deletions on the IRGSP rice pseudomolecules were classified as genic and intergenic, and locations within gene models were annotated. The number of SNPs, insertions, and deletions in each class is shown.

### Relationship between Cultivars by Comparison of SNPs

The variation among cultivars was investigated by assessing SNPs and InDels across individual chromosomes (Table 3). In *temperate japonica* cultivars, chromosomes 4 and 11 had high densities of SNPs and InDels, whereas chromosome 9 had low densities of variants in all cultivars, except Gohyakumangoku. SNP density across chromosome 9 in Gohyakumangoku was about 5 times higher than that in other *temperate japonica* cultivars. Some cultivars showed a characteristic pattern of SNP location on chromosomes. The precise origins of these differences in chromosomes were detected by comparing the total number of SNPs per megabase in the chromosomes of each cultivar (Figures S2–S13). There were 7 regions in which all cultivars shared high density, with over 500 SNPs/Mb; these included 24–25 Mb, 15–17 Mb, 15–16 Mb, 25–26 Mb, 2–3 Mb, 6–8 Mb, and 27–28 Mb on chromosomes 2, 3, 4, 7, 10, 11, and 12, respectively. In Moroberekan, several regions showed extremely high densities of SNPs, such as 8–13 Mb on chromosome 4, 6–10 Mb on chromosome 10, and 11–23 Mb on chromosome 12, whereas the densities of SNPs in these regions in the other cultivars were low. Our analysis approach was unable to detect regions with less than 50 SNPs/Mb in any of the 7 cultivars.

Comparison of SNPs and InDels indicated that the pair of cultivars Omachi and Yamadanishiki had the closest relationship among all the cultivars. However, there were differences in SNP distributions between these 2 cultivars at 27 regions, indicating a more than 5-fold difference in the number of SNPs. In particular, 7 regions showed marked differences, i.e. 8–9 Mb on chromosome 2, 2–5 Mb on chromosome 4, 0–2 Mb on chromosome 7, 9–11 Mb and 14–16 Mb on chromosome 8, 10–12 Mb on chromosome 9, and 19–20 Mb on chromosome 10. Between Omachi and the other cultivars, the number of these differing regions increased. In the cases of Kameji, Koshihikari, and Moroberekan, 55, 122, and 248 regions, respectively, were markedly different, again indicating a possible genomic relationship.

### Analyses of Unmapped Sequence Reads

In terms of CDSs, whole genome sequencing with a depth of 30× the genome provided over 80% coding sequences in 90% of genes; however, in each of the cultivar, there still remained many CDSs that were not covered by any short-reads. The unavailable sequences corresponding to these CDSs were expected to be present in the unmapped reads, which varied from 2.6–7.0% of the total reads in the 7 cultivars (Table S1). The unavailable sequences were reduced and annotated CDSs were increased by generating contigs by assembling unmapped reads and remapping these to the Nipponbare genome. The effect of read length on assembly quality was determined by preparing Omachi datasets containing different read lengths by trimming an original length of 74 bases. Among these trimmed reads, reads having quality value over 20 were used to decrease the rate of mis-assembly. The percentages of reads with such quality were increased with decreasing read length (Table S3). In the case of reads of original length, only 2% reads had such quality, but the trimmed 40 base reads contained 8.7% reads with such quality. In general, the sequence quality decreases with advanced read sequencing by using NGS; hence, filtering out the low-quality reads following trimming would decrease the rate of mis-assembly.

The quality of the assembly was shown in terms of output parameters in the VelvetOptimizer program and included the number of assembled reads; Velvet hash values; total number of contigs; N50 length; longest contig length; number of contigs longer than 1,000 bases; and total bases in contigs longer than 1,000 bases (Table S3). Significant trimming of the read length affected the number and length of contigs that could be constructed. Among these trimmed read lengths, reads with a length of 50 bases could generate the largest number and longest contigs in terms of N50 length. Under this condition, the total numbers of contigs that could be constructed were 6,903, which corresponded to approximately 80% of applied reads. The total length of all contigs was 2,479,877 bases. Application of the same approaches to other cultivars resulted in >2,500 contigs (Table S3). Notably, assembling of Moroberekan reads could yield over 20,000 contigs.

The resultant contigs were annotated to confirm that they were similar to the Nipponbare sequences. A sequence similarity search was conducted against the Nipponbare genome by using the BLAST algorithm, with an E value threshold of 10^−10^ [23]. Many contigs showed similarity to sequences in Nipponbare chromosomes 1–12. Of the total, 17–28% contigs were aligned to unique regions of the Nipponbare genome, and the remaining 47–71% contigs were aligned to multiple regions (Table 5). The degree of unique hit sequences in the contig sequences varied in cultivars. However, it reached over 80% in approximately 30% of contigs aligned on the unique regions in each of the cultivars (Figure S14). About 2–3% of contigs were aligned to about 1 Mb unanchored sequences, which were categorized to be Nipponbare sequences unaligned onto chromosomes 1–12.

**Table 5:** Annotation of contig sequences assembled from unmapped reads.

	Total	Nipponbare genome						No hit	(%)
		Chromosome 1–12				Unanchored	(%)		
		Unique	(%)	Multi	(%)				
Omachi	6,903	1,834	26.6	3,250	47.1	231	3.3	1,588	23.0
Yamadanishiki	4,020	827	20.6	2,785	69.3	63	1.6	345	8.6
Kameji	5,415	1,088	20.1	3,189	58.9	101	1.9	1,037	19.2
Gohyakumangoku	4,555	821	18.0	2,949	64.7	78	1.7	707	15.5
Koshihikari	3,773	809	21.4	2,482	65.8	60	1.6	422	11.2
Norin 8	2,554	434	17.0	1,802	70.6	42	1.6	276	10.8
Moroberekan	23,706	6,717	28.3	14,292	60.3	358	1.5	2,339	9.9

A similarity search of the contig sequences was conducted against the Nipponbare genome chromosomes 1–12 and unanchored sequences. The numbers in the categories of unique, multi-hits, and no hits show the total number of contigs classified.

The quality of contigs was validated by selecting and sequencing 12 Omachi contigs showing similarity to unique regions of the Nipponbare genome (Table S4). Among these, the sequences of 10 contigs were successfully amplified using primers shown in Table S4 and showed matches between Sanger and NGS sequences.

**Figure 2:**
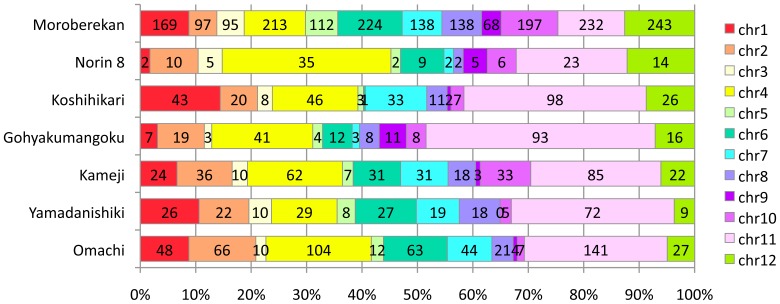
Configuration comparison of unique hit contigs among chromosomes. The numbers in the bars show the number of classified contigs.

The contigs, including with over 80% unique hit sequences, were distributed unevenly across the chromosomes (Figure 2). Specifically, more contigs mapped to chromosomes 4 and 11 compared to other chromosomes, except in Moroberekan, and fewest contigs mapped to chromosome 9, followed by chromosome 5. In Moroberekan, such contigs were distributed evenly across the chromosomes. Whether those contigs covered new gene regions was determined by first listing the regions that had many reference nucleotides without mapped short reads in each cultivar. Next, the contigs having unique hit sequences were mapped to listed regions. This approach allowed the identification of a total of 315 contigs containing newly mapped sequences that had not been covered by any short-reads (Table S5). The regions covered by these contigs coded for a total of 119 genes (Table 6, Table S6). A significant number of uniquely hit genes were found in Omachi and Moroberekan, but not in Norin 8. This might indicate that the method used was effective for distantly related cultivars from the reference cultivar. Thus, contig construction using the unmapped reads was very effective in obtaining information on CDSs.

**Table 6:** Number of newly mapped genes by contigs in the Nipponbare genome.

	Number of contigs		Number of genes in newly mapped regions
	Total	Newly mapped	
Omachi	547	75	28
Yamadanishiki	245	21	9
Kameji	362	15	4
Gohyakumangoku	225	13	7
Koshihikari	298	19	5
Norin 8	115	7	2
Moroberekan	1,926	165	64

The newly mapped contigs contained newly mapped sequences of Nipponbare that had not been covered by any short-reads.

The “no hit” contigs in Table 5 were annotated to the sequences of the other *japonica* rice cultivars by using BLASTn search with *Oryza sativa japonica* rice. In all, 22–249 contigs were assigned to a part of the sequences in the targets (Table 7). However, in this case, the size of the target was very large compared to that of the contig length. Among the contigs assigned, 7–87 contigs showed similarity with over 80% of the contig length to a part of the target sequences. The descriptions of targets are listed in Table S7. The most hit target was the locus “*Pik,*” which is known to confer rice blast resistance in cultivars of Tsuyuake and Kanto 51 *japonica* rice [24], [25].

**Table 7:** Numbers of contigs showing sequence similarity to Oryza sativa japonica and Oryza sativa indica.

	Hit		No hit
	*Japonica*	*Indica*	
Omachi	214	76	1,298
Yamadanishiki	57	11	277
Kameji	98	71	868
Gohyakumangoku	53	5	649
Koshihikari	75	6	341
Norin 8	22	10	244
Moroberekan	249	82	2,008

A similarity search of the contig sequences that were unaligned to regions of Nipponbare sequence was sequentially conducted against *Oryza sativa japonica* and *Oryza sativa indica* by using NCBI BLASTn search. The numbers in the categories of hit to *japonica* or *indica*, and no hit show the total number of contigs classified.

Next, the remaining contigs that did not show hits to the *japonica* rice sequences were searched using BLASTn with *Oryza sativa indica* rice. Considerable numbers of contigs showed hits to *indica* rice in all of the 7 cultivars (Table 7). Specifically, in Omachi, Kameji, and Moroberekan, which were older cultivars, and a *tropical japonica* cultivar, more contigs were aligned to *indica* rice genome. In Moroberekan, 3 contigs were assigned on a part of the sequences of Kasalath genome, of which the target contained the sequences corresponding to the phosphorus uptake 1 QTL of Nipponbare (Table S7) [26]. Thus, the expanding of the target cultivars resulted in increased annotation efficiency.

## Discussion

To increase the available genome information in rice, we selected and analysed the whole genomes of 6 different types of cultivars, namely, Yamadanishiki, Gohyakumangoku, Kameji, Koshihikari, Norin 8, and Moroberekan. Comparisons of the genomes of these cultivars, which differ in uses, breeding background, and cultivation area, would be expected to provide interesting and useful information.

Previously, we reported numerous polymorphisms between the Nipponbare reference IRGSP build4 and Omachi genome sequences [15]. Significant differences in the number of SNPs and InDels and their annotation have not detected between the results of IRGSP build4 and build5 (data not shown). However, the remarkable changes were observed in the number of SNPs and InDels of Omachi by IRGSP1.0 than in those by IRGSP build4 (Tables 1, 3, [15]). The genome sequence and the annotation data of IRGSP1.0 probably had significantly improved from the previous database. In this study, we used Nipponbare reference IRGSP1.0. Over 83% of the whole genome of each of the cultivars was successfully covered, and this 80% sequences comprised >90% of the total genes in each genome (Tables 1, 2). Thus, the Nipponbare genome sequence was useful as a reference for gene regions determined using NGS in various *japonica* rice genomes, including *tropical japonica* Moroberekan. Large numbers of SNPs and InDels were identified between each cultivar and the Nipponbare reference genome sequence (Table 3). The sequence data and polymorphisms are available at NGRC_Rice_Build 1.0 and are expected to be useful for genomic and genetic analyses.

In a previous report, many polymorphisms between the Nipponbare reference and Omachi genome sequences had been found with a sequencing depth of approximately 20× the genome [15]. This sequencing depth was sufficient to detect DNA polymorphisms that could be used as DNA markers. However, approximately 20% of the total number of genes had not yet been covered under this condition, and this was deemed insufficient coverage of CDSs in gene regions. When the relation between sequence coverage and sequencing depth was investigated, we found that the number of sequenced genes approached saturation with a sequencing depth of 30× the genome (Figure 1). Under this condition, >90% of the total genes in Omachi were accounted for among genes with >90% of CDSs covered. This indicated that increasing the sequencing depth was needed to sufficiently cover CDSs and is a promising approach to increase efficiency of identifying genes responsible for a phenotype.

Considering the sequence coverage of CDS regions of genes in each cultivar, CDSs of Koshihikari were covered almost completely, whereas those of Kameji were covered least well (Table 2). Interestingly, although the CDS regions of 1,188 Moroberekan genes were not covered by any short-reads (twice as many as those of the other *temperate japonica* cultivars), many genes with CDS regions covered completely were detected similar to that of Koshihikari. In Moroberekan, 39,181 genes were completely sequenced, which was more than that of other *japonica* cultivars (Table 2). This finding suggests that a majority of CDS regions in Moroberekan have close similarity to a reference, likely reflecting the concept that genic regions are better conserved than intergenic regions, and that high densities of polymorphisms are found in intergenic regions. Moroberekan results further indicated that the Nipponbare sequences could be used as a reference for cultivars having a different breeding background than that of *japonica* rice in Japan, such as Moroberekan. Applying NGS technology to elucidate the genomes of even more distantly related cultivars would yield interesting results.

Increased completion of a whole-genome sequence is preferable for genetic analyses. However, there were regions that could not be sequenced by the method used in this study. These regions varied among cultivars depending on the genetic distance from the reference sequence (Table 1). Because the sequenced short-reads were reconstructed on a reference sequence on the basis of homology between the genomes, the excluded regions are expected to include regions containing multiple SNPs, long insertions and deletions, repetitive regions, undefined bases in the reference genome, and sequences homologous to the chloroplast and mitochondrial genomes. Sequencing of the unavailable regions of multiple SNPs and InDels might be helpful in the identification of cultivar-specific gene functions, because some genes related to the characteristics of a given cultivar included them, for example, some disease-resistance genes containing leucine-rich repeat domains [24], [25]. In order to obtain complete sequence, we attempted to decrease the unmapped regions by assembling the unmapped short-reads (Table S5). This analysis resulted in a total 3,718 contigs containing unique and similar sequences with the Nipponbare genome. Unexpectedly, about 90% of these contigs were assigned to the regions covered by the original short read mapping. The reason why a majority of contigs assembled by unmapped short reads were mapped to known regions was not clear. One of the reasons for this might be the difference in the search criteria used for the short-read mapping and sequence similarity searching of contigs. If the unavailable reads had many SNPs and an incorrect base by error over an acceptable range, the resultant contigs might have sufficient sequence similarity to be aligned on similar regions. Actually, the number of gene sequences covered in the 7 cultivars could be increased using this approach. Newly covered sequences were detected in the 315 contigs, which contained a total of 119 new gene regions (Table S6). Especially, the number of these regions in Moroberekan was larger than that in *temperate japonica.* Interestingly, the genes, including the newly mapped sequences, tended to be enriched for kinase genes (Table S6) and contained a leucine-rich repeat domain that affords disease resistance in plants. High frequencies of SNPs have been reported in those genes from rice, Arabidopsis, and maize genomes [27]–[29]. Thus, construction of contigs using unmapped reads could cover gaps that remained by using original short-read mapping and might allow to facilitate gene identification.

More contigs were annotated by expanding the target of sequence similarity search from Nipponbare to other *japonica* and *indica* sequences. Many contigs were found to show hits to the rice blast resistance *Pik* locus with variation of sequences in different *japonica* cultivars [24], [25]. The sequence similarity search to *indica* rice cultivars revealed that, in the old cultivars Omachi and Kameji and *toropical japonica* cultivar Moroberekan, contigs aligned to sequences of *indica* rice were more than those in the other cultivars. This might be related their genetic distance from *indica* cultivars (Table 7). Further investigation is warranted to understand the biological function of hit sequences included in the contigs. However, contigs formed by assembling unmapped short reads might increase their value.

Our results suggested a genetic relationship among the 7 cultivars. There seemed to be an increasing tendency of coverage of uniquely mapped reads in the modern cultivars similar to Nipponbare. Old cultivars, Kameji and Omachi, and *tropical japonica* cultivar Moroberekan showed slightly low coverage (Table 1). The increased numbers of genes not covered and of polymorphism detected seemed to indicate the increasing distance from Nipponbare, where Kameji was an exception (Tables 2, 3). Actually, about 4–10-fold numbers of SNPs, insertions, and deletions were detected in Moroberekan. The number of unmapped reads also tended to increase in cultivars with increasing distance from Nipponbare (Table S1). In Omachi, Kameji, and Moroberekan, many contigs assembled from those unmapped reads showed similarity to *indica* rice cultivars (Table 7). These results suggested a genetic relationship among the cultivars.

In conclusion, in this study, we analysed the whole genomes of 6 different cultivars using NGS technology and obtained a total 1,503,704 SNPs and 368,504 InDels in *temperate* and *tropical japonica* cultivars by using Nipponbare genome as a reference. A public database of this genome information has been developed. Additionally, we defined 2 approaches that efficiently used reads obtained by NGS. First, by using the Omachi sequence data, we showed that a sequencing depth of 30× would be required to sufficiently cover CDSs to allow genetic analyses. Furthermore, we could improve mapping efficiency to obtain contigs by assembling unmapped reads, resulting in the detection of new genes. Our attempts might be helpful, for example, in increasing the value of NGS reads for detecting causal genes of particular phenotypes. Taken together, the sequence data and DNA polymorphism results obtained provide a basis for advancing research in genetic and genomic analyses of rice.

## Materials and Methods

### Library Construction and Sequencing

*Oryza sativa* L. cv. Yamadanishiki, Gohyakumangoku, Kameji, Koshihikari, Norin 8, and Moroberekan were used in this study. Yamadanishiki and Gohyakumangoku were obtained from a previous collection [30], [31]. Kameji JRC 31, Koshihikari JP: 80825, and Moroberekan JP: 14643 were obtained from the NIAS Genebank (http://www.gene.affrc.go.jp/index_j.php). Norin 8 was maintained as a laboratory stock.

Genomic DNA samples were extracted from 5 or 10 plants with Nucleon PhytoPure (GE Healthcare BioSciences, Little Chalfont, UK) and used for the preparation of sequencing libraries, according to the manufacturer’s protocols (Illumina, San Diego, CA, USA). Fragments of the libraries were paired-end sequenced using Genome Analyzer IIx and HiSeq2000 (Illumina). The length of all sequences generated was 74–100 nucleotides. NGS reads of these genomes have been submitted to DDBJ (http://www.ddbj.nig.ac.jp/index-e.html) under the following accession number DRA000897. The Omachi NGS reads were previously generated [15].

### Sequence Mapping and SNP/InDel Identification

Filtering rules were applied to select reads that were mapped to the rice chromosomal genome (*Oryza sativa* L. cv. Nipponbare, the Os-Nipponbare-Reference-IRGSP-1.0, http://rapdb.dna.affrc.go.jp/download/irgsp1.html, Rice Genome Sequencing Project, 2008) using ELAND (optional software for the Illumina GA pipeline system ver. 1.6–1.8). Mapping to the chromosomal and organelle genomes (*O. sativa* ssp. *japonica* group chloroplasts and Nipponbare mitochondria, DDBJ accession numbers X15901 and DQ167400, respectively) was performed using the Burrows–Wheeler alignment tool (BWA) software algorithm, allowing 2 mismatches from the 1^st^ to 35^th^ bases of the read [32]. BWA outputs were analysed using SAMtools software [33]. For SNP and InDel discovery, the algorithm implemented in SAMtools considered only reads that aligned to a unique location of the Nipponbare genome. Three filters were then applied to decrease the rate of false-positive SNPs and InDels: a target depth ≥5; target minimum base quality of 30; and call rate of a polymorphism >90% for SNPs and >30% for InDels. The physical positions of each sequence, including SNP and InDel information, were integrated into the annotated gene set of IRGSP1.0. SNPs and InDels in gene regions and other genomic regions were annotated as genic and intergenic, respectively. The genic SNPs and InDels were classified as CDS, UTR5′, and UTR3′ (UTRs), or other (introns), according to those positions. SNPs in the CDSs were separated into synonymous and non-synonymous (amino acid substitutions). These data of IRGSP1.0 are available at GBrowse [34], NGRC_Rice_Build1.0 (http://www.nodai-genome.org/oryza_sativa_en.html).

### Validation of SNPs and Contigs

An SNP array was designed using SNPs detected between Omachi and Nipponbare sequences by using BWA software [15]. Some SNPs detected between Omachi and Nipponbare sequences by MAQ ver. 0.6.6 [35] were added onto the array according to MAQ instructions. These SNPs between Omachi and Nipponbare, as well as the core SNPs among *japonica* rice [5], were used for validation using the Illumina Bead Station 500G genotyping system following the manufacturer’s instructions (Illumina). Assembled contigs were validated using an ABI 3730xl sequencer (Applied Biosystems, Tokyo, Japan) according to the manufacturer’s protocols.

### *De novo* Assemble of Unmapped Reads

Paired-end reads were used as unmapped reads after removing both-sided and/or one-sided reads assigned to the reference. The 3′-ends of unmapped reads were trimmed to various lengths. The rate of mis-assembly was reduced by using only those reads that had quality value of >20. Contigs were assembled from these trimmed reads using VelvetOptimizer ver. 2.2.0, a commonly used short-read assembler, by using automatic optimisation of parameter options (http://www.bioinformatics.net.au/software.velvetoptimiser.shtml).

## Supporting Information

Figure S1 **Distribution of the number of SNPs across different call rates.**
(PDF)

Figure S2 **(Caption applies for Figures S2-S13) Distribution of SNPs between the different cultivars and Nipponbare reference sequence across the 12 rice chromosomes.** The x-axis represents the physical distance along each chromosome, split into 1 Mb windows. The y-axis indicates the number of SNPs. The black arrows show the high-density regions common among the 7 cultivars. The blue arrows show the remarkably different regions between Omachi and Yamadanishiki.
(PDF)

Figure S3 (PDF)

Figure S4 (PDF)

Figure S5 (PDF)

Figure S6 (PDF)

Figure S7 (PDF)

Figure S8 (PDF)

Figure S9 (PDF)

Figure S10 (PDF)

Figure S11 (PDF)

Figure S12 (PDF)

Figure S13 (PDF)

Figure S14 **The degree of unique hit sequences in the contig sequences.** The results are shown by colour boxes classified to 5 groups on the basis of percentages of the alignment length in the contig length: 0–20% (blue), 20–40% (green), 40–60% (yellow), 60–80% (orange), and 80–100% (red). The numbers in the bars show the number of contigs.
(PDF)

Table S1 **Classification of short-reads mapped onto mitochondrial, plastid, and chromosomal genome IRGSP1.0, as well as unmapped reads.**
(PDF)

Table S2 **Validation of genome-wide SNPs by array.** Genomic DNAs of each cultivar and Nipponbare were analysed using SNP arrays by using the Illumina Bead Station 500G system. Omachi, Yamadanishiki, Kameji, Gohyakumangoku, Koshihikari, and Norin 8 were analyzed using a set of SNPs that was selected from SNP resources developed on the basis of Omachi and Nippobare. Moroberekan was analyzed using a set of SNPs that was selected from SNP resources developed on the basis of *japonica* rice cultivars.
(PDF)

Table S3 **Evaluation of assembly quality.** Assembly results from the unmapped reads trimmed to various lengths are shown. Those reads were assembled into contigs by using VelvetOptimizer.
(PDF)

Table S4 **Validation results of de novo assemblies.** +means validated contigs; blank means contigs not amplified with these primers.
(PDF)

Table S5 **List of contigs similar to unique regions in the Nipponbare reference genome.** Position and length of aligned regions in the Nipponbare genome sequence are shown from Target_start to Target_end, and bases. The number of bases that were not mapped to any region by reads is shown as depth = 0. Total base means the number of bases mapped onto the regions.
(XLSX)

Table S6 **Newly covered genes by contigs in the Nipponbare genome.**
(XLSX)

Table S7 **Number of contigs aligned to** ***japonica*** **and** ***indica*** **rice, including sequences showing over 80% hits.**
(XLSX)

## References

[1] Lee SH, van der Werf JH, Hayes BJ, Goddard ME, Visscher PM (2008) Predicting unobserved phenotypes for complex traits from whole-genome SNP data. PLoS Genet 4: e1000231. 18949033 10.1371/journal.pgen.1000231 PMC2565502

[2] Atwell S, Huang YS, Vilhjálmsson BJ, Willems G, Horton M, et al (2010) Genome-wide association study of 107 phenotypes in *Arabidopsis thaliana* inbred lines. Nature 465: 627–631. 20336072 10.1038/nature08800 PMC3023908

[3] Huang X, Wei X, Sang T, Zhao Q, Feng Q, et al (2010) Genome-wide association studies of 14 agronomic traits in rice landraces. Nat Genet 42: 961–967. 20972439 10.1038/ng.695

[4] Huang X, Kurata N, Wei X, Wang ZX, Wang A, et al (2012) A map of rice genome variation reveals the origin of cultivated rice. Nature 490: 497–501. 23034647 10.1038/nature11532 PMC7518720

[5] Nagasaki H, Ebana K, Shibaya T, Yonemaru J, Yano M (2010) Core single-nucleotide polymorphisms - a tool for genetic analysis of the Japanese rice population. Breed Sci 60: 648–655.

[6] Yamamoto T, Nagasaki H, Yonemaru J, Ebana K, Nakajima M, et al (2010) Fine definition of the pedigree haplotypes of closely related rice cultivars by means of genome-wide discovery of single-nucleotide polymorphisms. BMC Genomics 11: 267. 20423466 10.1186/1471-2164-11-267 PMC2874813

[7] Bachlava E, Taylor CA, Tang S, Bowers JE, Mandel JR, et al (2012) SNP discovery and development of a high-density genotyping array for sunflower. PLoS One 7: e29814. 22238659 10.1371/journal.pone.0029814 PMC3251610

[8] Gaur R, Azam S, Jeena G, Khan AW, Choudhary S, et al (2012) High-throughput SNP discovery and genotyping for constructing a saturated linkage map of chickpea (*Cicer arietinum* L.). DNA Res 19: 357–373. 22864163 10.1093/dnares/dss018 PMC3473369

[9] Poland JA, Brown PJ, Sorrells ME, Jannink JL (2012) Development of high-density genetic maps for barley and wheat using a novel two-enzyme genotyping-by-sequencing approach. PLoS One 7: e32253. 22389690 10.1371/journal.pone.0032253 PMC3289635

[10] Varshney RK, Nayak SN, May GD, Jackson SA (2009) Next-generation sequencing technologies and their implications for crop genetics and breeding. Trends Biotechnol 27: 522–530. 19679362 10.1016/j.tibtech.2009.05.006

[11] McCouch SR, Zhao K, Wright M, Tung C, Ebana K, et al (2010) Development of genome-wide SNP assays for rice. Breed Sci 60: 524–535.

[12] Kawahara Y, de la Bastide M, Hamilton JP, Kanamori H, McCombie WR, et al (2013) Improvement of the Oryza sativa Nipponbare reference genome using next generation sequence and optical map data. Rice 6: 4. 24280374 10.1186/1939-8433-6-4 PMC5395016

[13] Sakai H, Lee SS, Tanaka T, Numa H, Kim J, et al (2013) Rice Annotation Project Database (RAP-DB): an integrative and interactive database for rice genomics. Plant Cell Physiol 54: e6. 23299411 10.1093/pcp/pcs183 PMC3583025

[14] Yu J, Hu S, Wang J, Wong GK, Li S, et al (2002) A draft sequence of the rice genome (*Oryza sativa* L. ssp. *indica*). Science 296: 79–92. 11935017 10.1126/science.1068037

[15] Arai-Kichise Y, Shiwa Y, Nagasaki H, Ebana K, Yoshikawa H, et al (2011) Discovery of genome-wide DNA polymorphisms in a landrace cultivar of *Japonica* rice by whole-genome sequencing. Plant Cell Physiol 52: 274–282. 21258067 10.1093/pcp/pcr003 PMC3037082

[16] Lin Y, Li J, Shen H, Zhang L, Papasian CJ, et al (2011) Comparative studies of de novo assembly tools for next-generation sequencing technologies. Bioinformatics 27: 2031–2037. 21636596 10.1093/bioinformatics/btr319 PMC3137213

[17] Austin RS, Vidaurre D, Stamatiou G, Breit R, Provart NJ, et al (2011) Next-generation mapping of Arabidopsis genes. Plant J 67: 715–725. 21518053 10.1111/j.1365-313X.2011.04619.x

[18] Sabot F, Picault N, El-Baidouri M, Llauro C, Chaparro C, et al (2011) Transpositional landscape of the rice genome revealed by paired-end mapping of high-throughput re-sequencing data. Plant J 66: 241–246. 21219509 10.1111/j.1365-313X.2011.04492.x

[19] Polko JK, Temanni MR, van Zanten M, van Workum W, Iburg S, et al (2012) Illumina sequencing technology as a method of identifying T-DNA insertion loci in activation-tagged *Arabidopsis thaliana* plants. Mol Plant 5: 948–950. 22461665 10.1093/mp/sss022

[20] Zhao K, Wright M, Kimball J, Eizenga G, McClung A, et al (2010) Genomic diversity and introgression in *O. sativa* reveal the impact of domestication and breeding on the rice genome. PLoS One 5: e10780. 20520727 10.1371/journal.pone.0010780 PMC2875394

[21] Wang GL, Mackill DJ, Bonman JM, McCouch SR, Champoux MC, et al (1994) RFLP mapping of genes conferring complete and partial resistance to blast in a durably resistant rice cultivar. Genetics 136: 1421–1434. 7912216 10.1093/genetics/136.4.1421 PMC1205921

[22] Zouzou M, Kouakou TH, Koné M, Issaka S (2008) Screening rice (*Oryza sativa* L.) varieties for resistance to rice yellow mottle virus. Scientific Research and Essay 3: 416–424.

[23] Altschul SF, Gish W, Miller W, Myers EW, Lipman DJ (1990) Basic local alignment search tool. J Mol Biol 215: 403–410. 2231712 10.1016/S0022-2836(05)80360-2

[24] Ashikawa I, Hayashi N, Yamane H, Kanamori H, Wu J, et al (2008) Two adjacent nucleotide-binding site-leucine-rich repeat class genes are required to confer *Pikm*-specific rice blast resistance. Genetics 180: 2267–2276. 18940787 10.1534/genetics.108.095034 PMC2600957

[25] Ashikawa I, Hayashi N, Abe F, Wu J, Matsumoto T, et al (2012) Characterization of the rice blast resistance gene *Pik* cloned from Kanto51. Mol Breeding 30: 485–494.

[26] Heuer S, Lu X, Chin JH, Tanaka JP, Kanamori H, et al (2009) Comparative sequence analyses of the major quantitative trait locus *p*hosphorus *up*take 1 (*Pup*1) reveal a complex genetic structure. Plant Biotechnol J 7: 456–457. 19422603 10.1111/j.1467-7652.2009.00415.x

[27] Clark RM, Schweikert G, Toomajian C, Ossowski S, Zeller G, et al (2007) Common sequence polymorphisms shaping genetic diversity in *Arabidopsis thaliana* . Science 317: 338–342. 17641193 10.1126/science.1138632

[28] McNally KL, Childs KL, Bohnert R, Davidson RM, Zhao K, et al (2009) Genomewide SNP variation reveals relationships among landraces and modern varieties of rice. Proc Natl Acad Sci USA 106: 12273–12278. 19597147 10.1073/pnas.0900992106 PMC2718348

[29] Lai J, Li R, Xu X, Jin W, Xu M, et al (2010) Genome-wide patterns of genetic variation among elite maize inbred lines. Nat Genet 42: 1027–1030. 20972441 10.1038/ng.684

[30] Yoshida S, Ikegami M, Kuze J, Sawada K, Hashimoto Z, et al (2002) QTL analysis for plant and grain characters of sake-brewing rice using a doubled haploid population. Breed Sci 52: 309–317.

[31] Hashimoto Z, Mori N, Kawamura M, Ishii T, Yoshida S, et al (2004) Genetic diversity and phylogeny of Japanese sake-brewing rice as revealed by AFLP and nuclear and chloroplast SSR markers. Theor Appl Genet 109: 1586–1596. 15375619 10.1007/s00122-004-1794-6

[32] Li H, Durbin R (2009) Fast and accurate short-read alignment with Burrows-Wheeler Transform. Bioinformatics 25: 1754–1760. 19451168 10.1093/bioinformatics/btp324 PMC2705234

[33] Li H, Handsaker B, Wysoker A, Fennell T, Ruan J, et al (2009) The Sequence Alignment/Map format and SAMtools. Bioinformatics 25: 2078–2079. 19505943 10.1093/bioinformatics/btp352 PMC2723002

[34] Stein LD, Mungall C, Shu S, Caudy M, Mangone M, et al (2002) The generic genome browser: a building block for a model organism system database. Genome Res 12: 1599–1610. 12368253 10.1101/gr.403602 PMC187535

[35] Li H, Ruan J, Durbin R (2008) Mapping short DNA sequencing reads and calling variants using mapping quality scores. Genome Res 18: 1851–1858. 18714091 10.1101/gr.078212.108 PMC2577856

