# Supplementary figures and images for: Genome-Wide DNA Polymorphisms in Seven Rice Cultivars of Temperate and Tropical Japonica Groups
Source: PLoS One. 2014 Jan 21;9(1):e86312. doi: 10.1371/journal.pone.0086312 (PMC3897683; doi:10.1371/journal.pone.0086312)

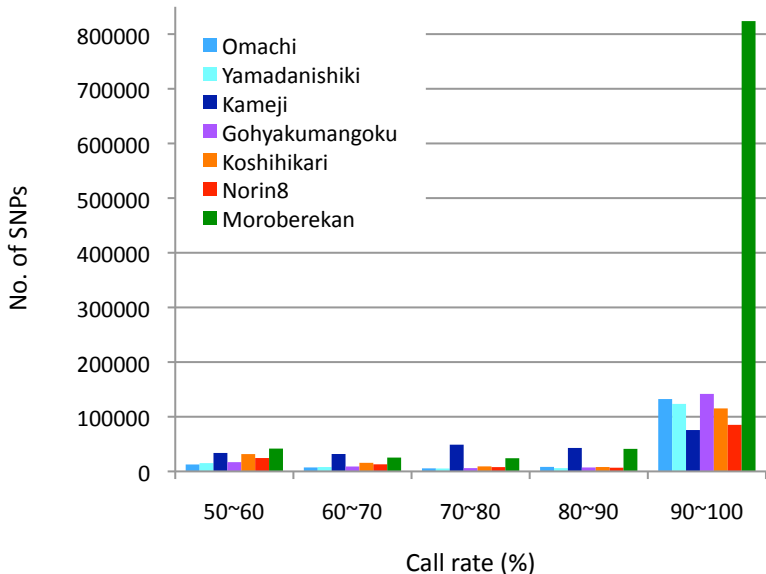

**Figure S1. Distribution of the number of SNPs across different call rates.**

Supplement: Figure S1 — Distribution of the number of SNPs across different call rates. (PDF) [file pone.0086312.s001.pdf]
