# Supplementary material for: Genome-Wide DNA Polymorphisms in Seven Rice Cultivars of Temperate and Tropical Japonica Groups
Source: PLoS One. 2014 Jan 21;9(1):e86312. doi: 10.1371/journal.pone.0086312 (PMC3897683; doi:10.1371/journal.pone.0086312)
Supplement: Figure S5 — (PDF) [file pone.0086312.s005.pdf]

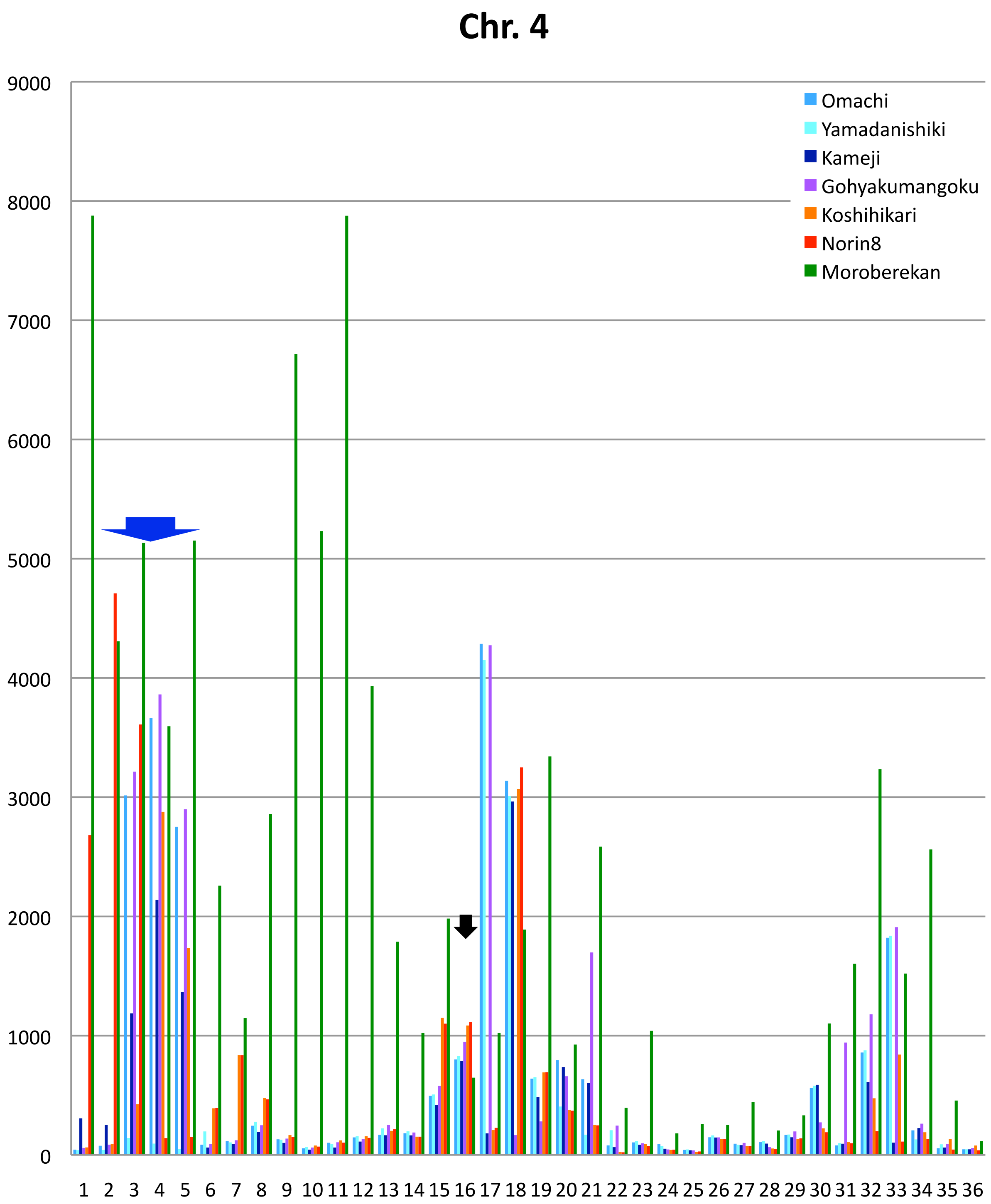

Figure S5. Distribution of SNPs between the different cultivars and Nipponbare reference sequence across the chromosome 4. The x-axis represents the physical distance along each chromosome, split into 1 Mb windows. The y-axis indicates the number of SNPs. The black arrows show the high-density regions common among the 7 cultivars. The blue arrows show the remarkably different regions between Omachi and Yamadanishiki.
