# Supplementary material for: Genome-Wide DNA Polymorphisms in Seven Rice Cultivars of Temperate and Tropical Japonica Groups
Source: PLoS One. 2014 Jan 21;9(1):e86312. doi: 10.1371/journal.pone.0086312 (PMC3897683; doi:10.1371/journal.pone.0086312)
Supplement: Figure S14 — The degree of unique hit sequences in the contig sequences. The results are shown by colour boxes classified to 5 groups on the basis of percentages of the alignment length in the contig length: 0–20% (blue), 20–40% (green), 40–60% (yellow), 60–80% (orange), and 80–100% (red). The numbers in the bars show the number of contigs. (PDF) [file pone.0086312.s014.pdf]

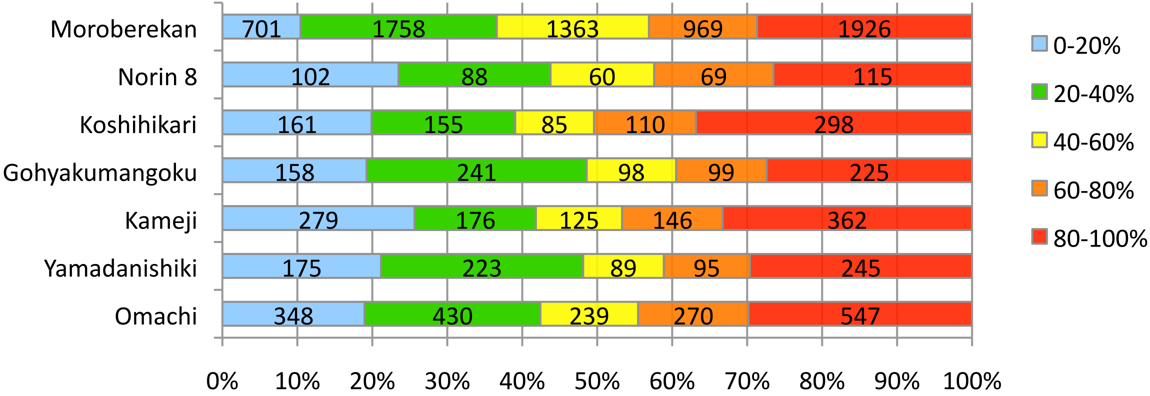

Figure S14. The degree of unique hit sequences in the contig sequences. The results are shown by colour boxes classified to 5 groups on the basis of percentages of the alignment length in the contig length: 0–20% (blue), 20–40% (green), 40–60% (yellow), 60–80% (orange), and 80–100% (red). The numbers in the bars show the number of contigs.
