# Supplementary material for: Genome-Wide DNA Polymorphisms in Seven Rice Cultivars of Temperate and Tropical Japonica Groups
Source: PLoS One. 2014 Jan 21;9(1):e86312. doi: 10.1371/journal.pone.0086312 (PMC3897683; doi:10.1371/journal.pone.0086312)
Supplement: Table S1 — Classification of short-reads mapped onto mitochondrial, plastid, and chromosomal genome IRGSP1.0, as well as unmapped reads. (PDF) [file pone.0086312.s015.pdf]

Table S1. Classification of short-reads mapped onto mitochondrial, plastid, and chromosomal genome IRGSP1.0, as well as unmapped reads.

	Mapped Reads												unmapped reads	
	Mitochondrion				Plastid				Chromosome					
	Unique		Multi		Unique		Multi		Unique		Multi			
	No. of reads	%	No. of reads	%	No. of reads	%	No. of reads	%	No. of reads	%	No. of reads	%	No. of reads	%
Omachi	2,465,440	0.8	1,908,084	0.6	6,546,757	2.2	3,845,886	1.3	230,205,002	77.5	39,627,132	13.3	12,613,864	4.2
Yamadanishiki	1,129,087	0.6	773,402	0.4	1,921,946	1.1	1,140,563	0.6	148,367,298	82.1	21,585,116	11.9	5,754,116	3.2
Kameji	2,929,666	1.3	2,333,624	1.0	12,811,248	5.8	5,132,130	2.3	167,729,858	75.3	24,176,161	10.9	7,502,681	3.4
Gohyakumangoku	934,816	0.4	696,109	0.3	2,494,812	1.0	1,242,848	0.5	198,189,791	83.1	26,123,664	10.9	8,921,475	3.7
Koshihikari	3,691,560	1.7	2,935,079	1.4	15,369,048	7.1	6,894,827	3.2	158,014,758	72.8	21,489,667	9.9	8,769,487	4.0
Norin 8	3,010,865	1.0	2,313,309	0.8	10,868,882	3.6	4,752,677	1.6	239,862,602	79.1	34,662,053	11.4	7,790,795	2.6
Moroberekan	2,759,796	1.6	2,348,358	1.4	8,790,353	5.2	5,631,926	3.3	118,052,502	69.7	20,053,332	11.8	11,777,888	7.0
