# Supplementary material for: Genome-Wide DNA Polymorphisms in Seven Rice Cultivars of Temperate and Tropical Japonica Groups
Source: PLoS One. 2014 Jan 21;9(1):e86312. doi: 10.1371/journal.pone.0086312 (PMC3897683; doi:10.1371/journal.pone.0086312)
Supplement: Table S2 — Validation of genome-wide SNPs by array. Genomic DNAs of each cultivar and Nipponbare were analysed using SNP arrays by using the Illumina Bead Station 500G system. Omachi, Yamadanishiki, Kameji, Gohyakumangoku, Koshihikari, and Norin 8 were analyzed using a set of SNPs that was selected from SNP resources developed on the basis of Omachi and Nippobare. Moroberekan was analyzed using a set of SNPs that was selected from SNP resources developed on the basis of japonica rice cultivars. (PDF) [file pone.0086312.s016.pdf]

Table S2. Validation of genome-wide SNPs by array.

Cultivar	Matched	Mismatched	Total
Omachi	535	0	535
Yamadanishiki	384	0	384
Kameji	296	0	296
Gohyakumangoku	316	0	316
Koshihikari	184	0	184
Norin8	152	1	153
Moroberekan	220	1	221

Genomic DNAs of each cultivar and Nipponbare were analysed using SNP arrays by using the Illumina Bead Station 500G system. Omachi, Yamadanishiki, Kameji, Gohyakumangoku, Koshihikari, and Norin 8 were analyzed using a set of SNPs that was selected from SNP resources developed on the basis of Omachi and Nippobare. Moroberekan was analyzed using a set of SNPs that was selected from SNP resources developed on the basis of *japonica* rice cultivars.
