# Supplementary material for: Genome-Wide DNA Polymorphisms in Seven Rice Cultivars of Temperate and Tropical Japonica Groups
Source: PLoS One. 2014 Jan 21;9(1):e86312. doi: 10.1371/journal.pone.0086312 (PMC3897683; doi:10.1371/journal.pone.0086312)
Supplement: Table S3 — Evaluation of assembly quality. Assembly results from the unmapped reads trimmed to various lengths are shown. Those reads were assembled into contigs by using VelvetOptimizer. (PDF) [file pone.0086312.s017.pdf]

Table S3. Evaluation of assembly quality.

Cultivar	Contig length (base)	Total number of sequences	Filtrated unmapped reads	Velvet hash value	Total number of used sequences	Total number of contigs	n50	length of longest contig	Total bases in contigs	Number of contigs > 1k	Total bases in contigs > 1k
Omachi	40	8,559,356	747,316	23	522,902	6,054	622	4,213	2,069,762	391	576,524
Omachi	50	8,559,356	544,564	27	427,684	6,903	621	4,498	2,479,877	466	670,098
Omachi	60	8,559,356	377,384	31	297,983	5,928	592	4,427	2,199,039	367	522,954
Omachi	74	8,559,356	176,936	37	133,061	4,162	526	4,223	1,531,336	216	307,434
Yamadanishiki	50	4,348,564	1,826,614	29	1,566,807	4,020	2,647	19,556	3,587,102	933	2,758,616
Kameji	50	5,289,498	2,559,720	31	2,232,138	5,415	2,283	28,450	4,286,839	1,162	3,173,808
Gohyakumangoku	50	7,067,046	2,287,502	31	2,002,998	4,555	2,861	20,605	4,189,891	1,048	3,269,311
Koshihikari	50	7,118,212	4,808,504	29	2,108,905	3,773	3,238	24,023	3,520,181	861	2,822,335
Norin 8	50	5,202,190	2,344,516	31	2,131,843	2,554	3,178	20,130	2,909,386	752	2,393,298
Moroberekan	50	8,981,714	4,613,662	31	3,775,044	23,706	822	9,612	10,896,353	2,613	4,602,860

Assembly results from the unmapped reads trimmed to various lengths are shown. Those reads were assembled into contigs by using VelvetOptimizer.
