# Supplementary material for: Genome-Wide DNA Polymorphisms in Seven Rice Cultivars of Temperate and Tropical Japonica Groups
Source: PLoS One. 2014 Jan 21;9(1):e86312. doi: 10.1371/journal.pone.0086312 (PMC3897683; doi:10.1371/journal.pone.0086312)
Supplement: Table S4 — Validation results of de novo assemblies. +means validated contigs; blank means contigs not amplified with these primers. (PDF) [file pone.0086312.s018.pdf]

Table S4. Validation results of *de novo* assemblies.

Contig ID	5’ primer	3’ primer	Alingment
NODE_162	tgggtcaatgcacatac	gccgaatcttgtggatt	+
NODE_201	tatcccagcaagcctttt	ccgagtaaggttaccaac	+
NODE_492	tagagtcattcaacctcc	agctattaaaagagagg	
NODE_681	gacatgcggctacattgt	tccttcacacactcaag	+
NODE_727	aaccatgggcatcatac	aatttgttgcctgcgtg	+
NODE_736	tttctttggcccgttgcct	gattgttctcgcgaaga	+
NODE_956	cagatgagccgtgttat	tgggagctgcagtattt	+
NODE_1127	gcttacactcccccatgt	ctggagaaaacacattatg	+
NODE_1660	atgtcggtatttgtttg	agatacaagtcggtacag	+
NODE_3333	agaaggtccaagtttgc	atgggtgtaatttactc	
NODE_4353	gatagattgtgaagtgtaaac	ttggtagactggaaagg	+
NODE_7043	acaacgtgcgcaggttcat	ggtggatcattgaatcccct	+

+ means validated contigs; blank means contigs not amplified with these primers.
